# Pilonidal sinus of the perianal region: Difficult to diagnose

**DOI:** 10.1016/j.ijscr.2020.05.071

**Published:** 2020-06-06

**Authors:** Ozlem Zeliha Sert

**Affiliations:** University of Health Science, Istanbul Haydarpasa Education and Research Hospital, Department of General Surgery Istanbul, Turkey

**Keywords:** Abscess, Perianal gland, Phenol, Pilonidal sinus

## Abstract

**Background:**

Pilonidal sinus usually arises in the area of natal cleft in young men. Hair accumulation in the wound in the perianal region is very rare. Although pelvic magnetic resonance imaging (MRI) is used in the differential diagnosis of perianal region diseases, it is not possible to diagnose perianal pilonidal sinus by MRI.

**Case:**

A 24-year-old male patient presented with the complaint of swelling and itching in the anus for several months. On physical examination, the area giving endure and fluctuation was detected in the perianal region. An area of ​​2 × 2 cm hyperintense in T2 was seen in pelvic MRI. In the surgical exploration, collection of hair was seen at 7 o’clock at perianal region with pus discharge. After the cavity was cleaned, crystallized phenol was applied to the area and the wound was left to secondary healing. Granulation was observed after 4 weeks.

**Conclusion:**

Perianal pilonidal sinus is very rare in the literature and is often confused with other perianal diseases such as perianal abscess or anal fistula. In this respect, when examining perianal diseases, especially in young men, perianal pilonidal sinus should be kept in mind in the differential diagnosis.

## Highlights

• Perianal pilonidal disease is quite rare all over the world.
• Differential diagnoses should be kept in mind in terms of perianal region diseases.
• Unusual surgical management of the perianal pilonidal sinus was demonstrated.
• This case report contributes to literature in terms of algorithm of perianal pilonidal sinus.

## 1 Introduction

The name pilonidal sinus was first described by Hodges in 1880. It is defined as a granulomatous lesion with dense hair, usually located on the sacrum in young men with hair [1]. Later, definition of pilonidal disease modified as, an acute or chronic infection in the subcutaneous fatty tissue, mainly in the natal (intergluteal) cleft. Diagnosis may be confused with anal fistula or hidradenitis suppurativa [2]. Here, we aimed to present our anal pilonidal sinus case which is very rare in the literature and presented only as case series.

## 2 Case

**Fig. 1:**
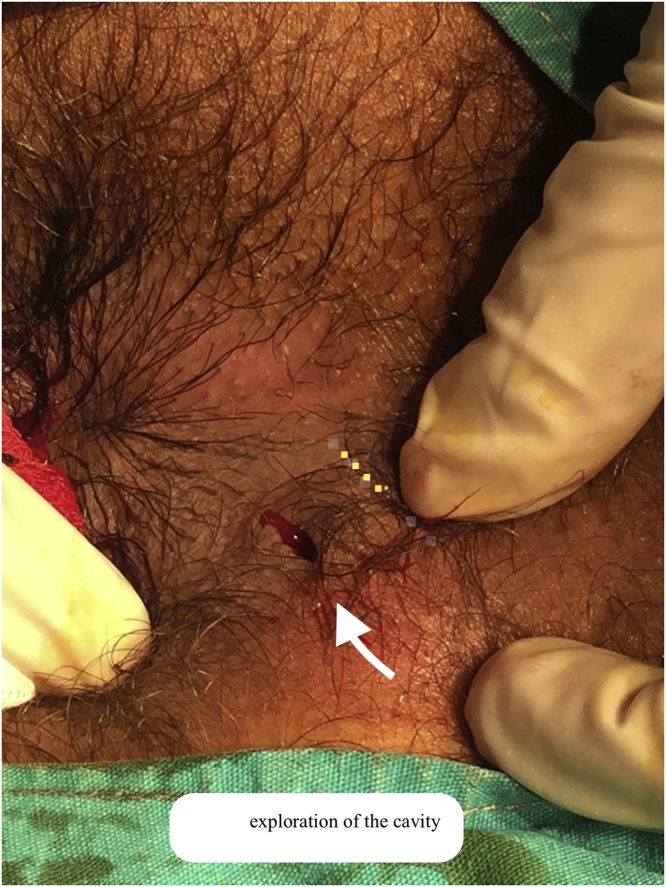
Exploration of the perianal pilonidal sinus in the prone position at 7 o’clock.

**Fig. 2:**
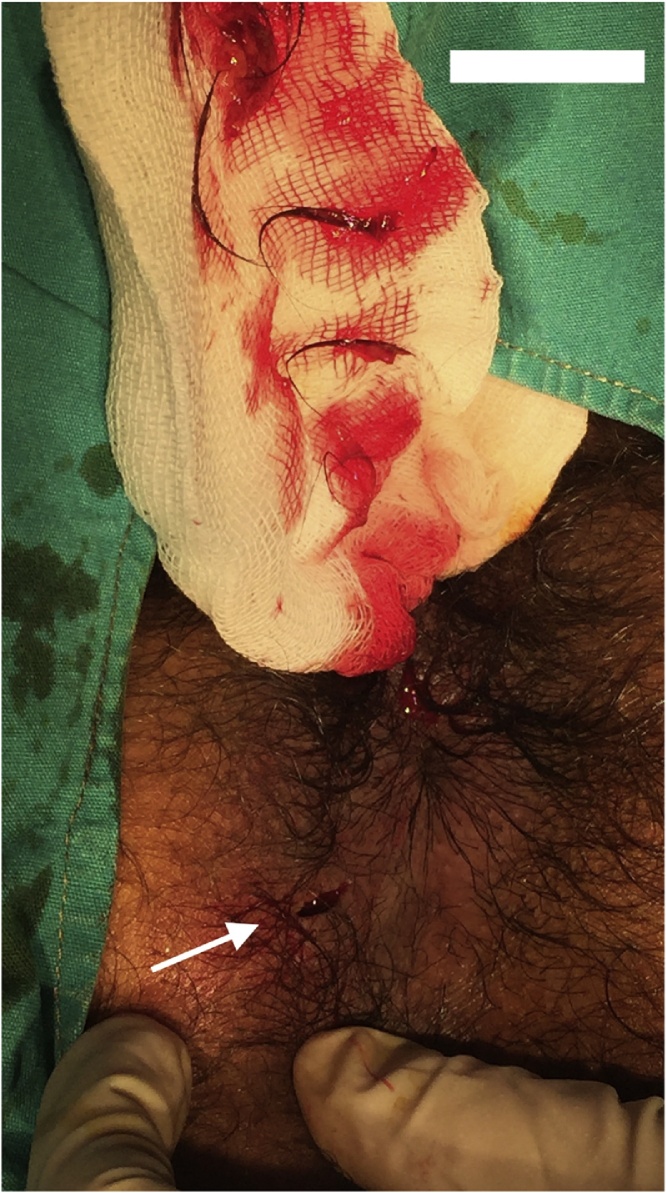
Removal of the hair from the cavity in the prone position at 7 o’clock.

**Fig. 3:**
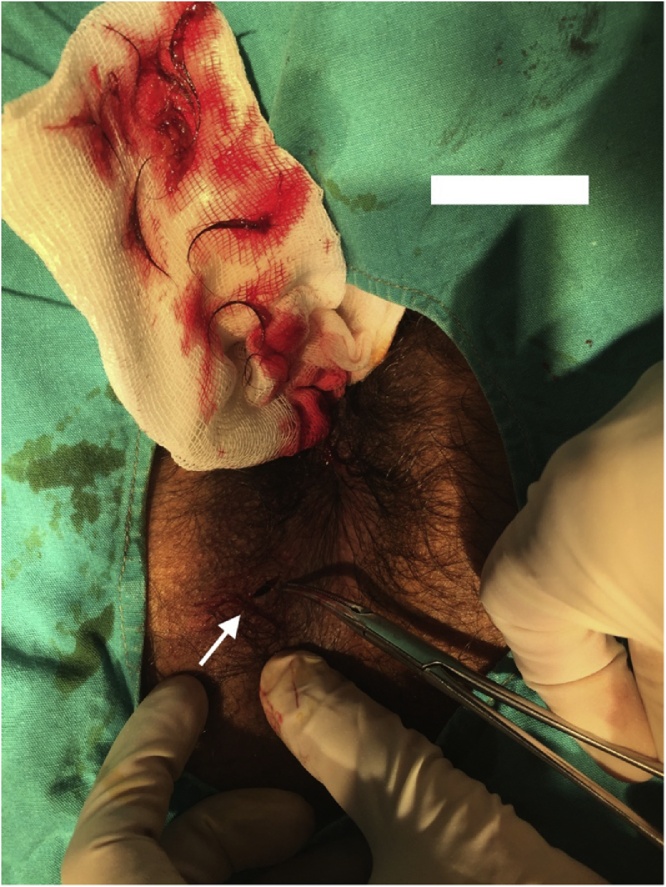
Removal of the hair and curation of the cavity.

A 24 year-old young male patient applied to the clinic with a 2-month history of itching and swelling around the anus in 2018. A physical examination in the prone position showed presence of left sided indurated and fluctuated approximately 2 × 2 cm diameter area of perianal region. He had no history of any previous anal surgery. MRI demonstrated that 2 × 2 cm [[1], [2], [3], [4], [5], [6], [7], [8]] hyperintense lesion in the posterior anal wall on T2 weighted images. In the surgical exploration, collection of hair was seen at 7 o’clock at perianal region with pus discharge (Fig. 1, Fig. 2, Fig. 3). The wall of the cavity curated and washed with saline solution. The cavity was blunt and had no association with internal or external sphincter. After irrigation of the cavity, crystallized phenol(Bota Farma İlaç Medikal İtriyat Kimya San. Tic. Ltd. Şti, Ankara, Turkey) applied to the cavity. The wound was left secondary healing. After 4 weeks, granulation was completed. Furthermore, he had no recurrent complaint.

## 3 Discussion

Pilonidal sinus typically derives in the space of natal cleft in young men. Diagnosis may be mixed up with the other perianal diseases like as perianal fistula or hidradenitis suppurativa. Pilonidal sinuses are usually present in sacrococcygeal area of young men. The presence of pilonidal sinus in association with anal canal is a quite rare situation. Less than 20 perianal pilonidal sinus have previously been reported [3].

Aggarwal K et al. presented the case of pilonidal sinus arose in the intersphincteric area in the anal canal [3]. In this case, the pilonidal sinus was located in the perianal region and had no association with sphincters.

Although there were similarities between the scheme of 3 cases published by T. H. Walsh and C. V. Mann in 1983 and our case, anal fistula did not develop in our case, and its location was suprasphincteric, not intersphincteric. On the other hand, Doll D et al. reported that when the anal canal was opened in their case, they encountered a very small amount of hair under the anal mucosa and submucosa [4].

Moreover, the etiopathogenesis of this case was more like anal abscess formation based on cryptoglandular theory. In the literature, several cases have been reported that presented with complicated anal fistula, a complication of anal abscess, and the etiology of which was found to be anal pilonidal sinus during surgery [[5], [6], [7]]. In our case, there was a risk of developing anal fistula, as well. However, no complications developed during long-term follow-up.

This case demonstrated a suprasphincteric located perianal pilonidal sinus abscess. In the treatment, a surgical approach such as the approach to anal abscess was applied, the abscess was drained, the hair in the cavity was removed, the cavity was washed with saline and crystallized phenol was applied to the cavity. Today, although the application of crystallized phenol to pilonidal sinus cases located in the sacrococcygeal area is common in the literature, no post-drainage phenol application was observed in the literature, similar to this case [8].

The limitation of this case report is that the patient's pictures after granulation have not been presented.

## 4 Conclusion

Anal pilonidal sinus is a very rare condition in clinical practice. When diagnosed, abscesses such as perianal abscess should be drained and the cavity should be cleaned and the wound should be left to secondary healing. However, as seen in this case, successful results can be obtained with crystallized phenol application after cleaning the cavity. We predict that with the spread of perianal pilonidal sinus cases in the literature, it will be easier to determine the treatment algorithm of perianal region diseases that are difficult to manage.

## Declaration of Competing Interest

The author has no conflict of interest.

## Sources of funding

None.

## Ethical approval

The study is exempt from ethical approval.

## Consent

Written informed consent was obtained from the patient for publication of this case report and accompanying images. A copy of the written consent is available for review by the Editor-in-Chief of this journal on request.

## Author contribution

**Table:** 

Author	Contributions	Disclosures
Sert OZ	Designed the study, wrote project, and drafted and revised the manuscript.	None to declare

## Registration of research studies

1 Name of the registry: not applicable.
2 Unique identifying number or registration ID: not applicable.
3 Hyperlink to your specific registration (must be publicly accessible and will be checked):not applicable.

## Guarantor

Ozlem Zeliha Sert, MD, Haydarpasa Education and Research Hospital, General Surgery Department, Istanbul, Turkey.

## Provenance and peer review

Not commissioned, externally peer-reviewed.
